# Radial Extracorporeal Shock Wave Therapy for Lower‐Limb Spasticity in an Individual With Subacute Stroke: A Case Report

**DOI:** 10.1002/ccr3.71942

**Published:** 2026-01-30

**Authors:** Daisuke Kato, Satoshi Hirano, Naoki Mori, Shota Itoh, Toshiki Ito, Taiki Yokote, Hirofumi Maeda, Yohei Otaka

**Affiliations:** ^1^ Department of Rehabilitation Fujita Health University Hospital Toyoake Japan; ^2^ Department of Rehabilitation Medicine School of Medicine, Fujita Health University Toyoake Japan; ^3^ Department of Well‐Being and Rehabilitation School of Medicine, Fujita Health University Toyoake Japan

**Keywords:** case report, modified Ashworth scale, muscle spasticity, radial extracorporeal shock wave therapy, range of motion, rehabilitation

## Abstract

Radial extracorporeal shock wave therapy showed immediate spasticity reduction and sustained range of motion improvement in a patient with subacute stroke. However, twice‐weekly sessions were insufficient for lasting spasticity control, suggesting that more frequent treatments may be required. Further research is needed to optimize treatment protocols.

## 1 Introduction

The treatment of spasticity as a sequela of stroke is a critical issue. Spasticity is a component of the upper motor neuron syndrome, a movement disorder characterized by a velocity‐dependent increase in tonic stretch reflexes (muscle tone) with exaggerated tendon jerks, resulting from hyperexcitability of the stretch reflex [1]. Stroke‐induced damage to the pyramidal and extrapyramidal tracts is thought to inhibit spinal reflexes and cause spasticity [2]. Non‐neurogenic changes resulting from muscle immobility caused by motor paralysis also increase muscle viscoelasticity, which in turn enhances spasticity [3]. The prevalence of spasticity in individuals with stroke ranges from 4% to 43%, making it a common sequela of stroke [4]. Spasticity contributes to reduced motor function, pain, and a decline in activities of daily living [5]. In particular, spasticity of the triceps surae muscle can cause equinus foot, increase instability during the stance phase, and reduce foot clearance during the swing phase, thereby increasing the risk of falls [6, 7]. Therefore, the treatment of triceps surae muscle spasticity is an important critical issue.

To date, the effectiveness of spasticity treatments has been primarily reported for injection‐based therapies such as botulinum toxin, motor point block with phenol, and intrathecal baclofen pump therapy [8, 9, 10, 11]. Botulinum toxin therapy, the gold standard, has demonstrated efficacy in systematic reviews and meta‐analyses, and its use is recommended in various guidelines [8, 9, 12]. However, it is an injection treatment that requires specialized medical skills and can be physically and psychologically demanding [13].

In contrast, extracorporeal shock wave therapy has gained attention as a non‐invasive and safe treatment option. Systematic reviews and meta‐analyses have demonstrated that extracorporeal shock wave therapy can improve spasticity [14]. This therapy is classified into two types based on the energy propagation pattern: focused and radial [15]. The radial type has been reported to be more effective than the focused type in treating spasticity in individuals with stroke [15]. A randomized controlled trial (RCT) of radial extracorporeal shock wave therapy (rESWT) applied to the triceps surae muscle in individuals with chronic stroke reported that improvements in spasticity lasted up to 8 weeks post‐treatment [15]. However, another RCT found that although spasticity improvement was observed immediately after treatment, the effect did not persist for 4 weeks post‐treatment, making the duration of the effect controversial [16]. Furthermore, most of the aforementioned systematic reviews and meta‐analyses were conducted in individuals in the chronic phase, and their effectiveness in those with subacute stroke remains unclear [14]. Additionally, there is significant variability in treatment protocols across studies, and a standard protocol has not yet been established [17]. One RCT examining the impact of treatment frequency on spasticity improvement reported that repeated treatments enhanced the therapeutic effect [18], but the carryover and cumulative effects of repeated treatments are not well understood. Moreover, no studies have detailed how outcomes, including spasticity, evolve over time in individuals with subacute stroke.

The purpose of this case report is to apply an rESWT treatment protocol, based on a previously validated protocol shown to be effective in improving spasticity in individuals with hemiplegia following chronic stroke, to an individual with hemiplegia following subacute stroke and to present the time‐course changes in triceps surae muscle spasticity and other outcomes before and after four rESWT sessions.

## 2 Case History

A 71‐year‐old man was admitted to a university hospital with a right thalamic hemorrhage. Prior to admission, he was fully independent in activities of daily living, despite having comorbid diabetes mellitus and postherpetic neuralgia. Written informed consent was obtained from the individual for publication of this case report, and the study complied with the CARE guidelines.

## 3 Investigations and Treatment

**FIGURE 1:**
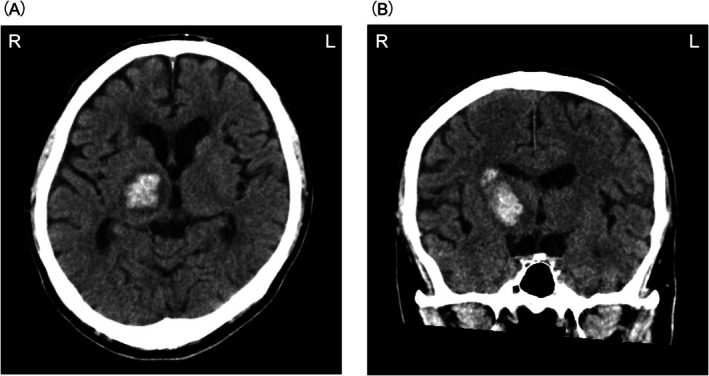
Computed tomography images of the brain at admission. (A) Horizontal plane. (B) Frontal plane. Hemorrhage in the right thalamus.

On admission, the individual was fully conscious but exhibited severe left hemiparesis and moderate stroke severity, as indicated by a total NIHSS score of 13. His activities of daily living were assessed at 39 points using the Functional Independence Measure (FIM), with 18 points for motor items and 21 points for cognitive items (Table 1). Computed tomography scans of the head revealed a 10.4 mL hemorrhage in the right thalamus (Figure 1). The individual received antihypertensive treatment with calcium channel blockers.

**TABLE 1:** Individual information at admission, pre‐treatment, post‐treatment, and discharge.

	Admission	Pre‐treatment	Post‐treatment	Discharge
GCS				
Eye opening	4	4	4	4
Verbal response	5	5	5	5
Motor response	6	6	6	6
SIAS motor LE				
Hip flexion	1	1	2	2
Knee extension	0	1	2	2
Footpad	0	0	1	1
ROM (°)				
Ankle dorsiflexion	20	10	20	20
MAS				
Triceps surae muscle	0	1+	1+	1+
FIM				
Walk item	1	1	1	4
Subtotal of motor domain	18	38	38	58
Subtotal of cognitive domain	21	21	24	30
Total	39	59	62	88

Abbreviations: FIM, functional independence measure; GCS, Glasgow Coma scale; MAS, modified Ashworth scale; ROM, range of motion; SIAS motor LE, Stroke impairment assessment set motor lower extremity total score.

Rehabilitation treatment was initiated on the first day after onset. At the start of rehabilitation, the triceps surae muscle tone was graded as 0 on the Modified Ashworth Scale (MAS), and the range of motion (ROM) of ankle dorsiflexion was not limited (Table 1). The individual began mobility exercises on the second day and gait training on the fourth day. Gait training required full assistance, using a knee–ankle–foot orthosis and a four‐point cane. On the fifth day, the individual was transferred to a convalescent inpatient rehabilitation ward for intensive rehabilitation.

**FIGURE 2:**
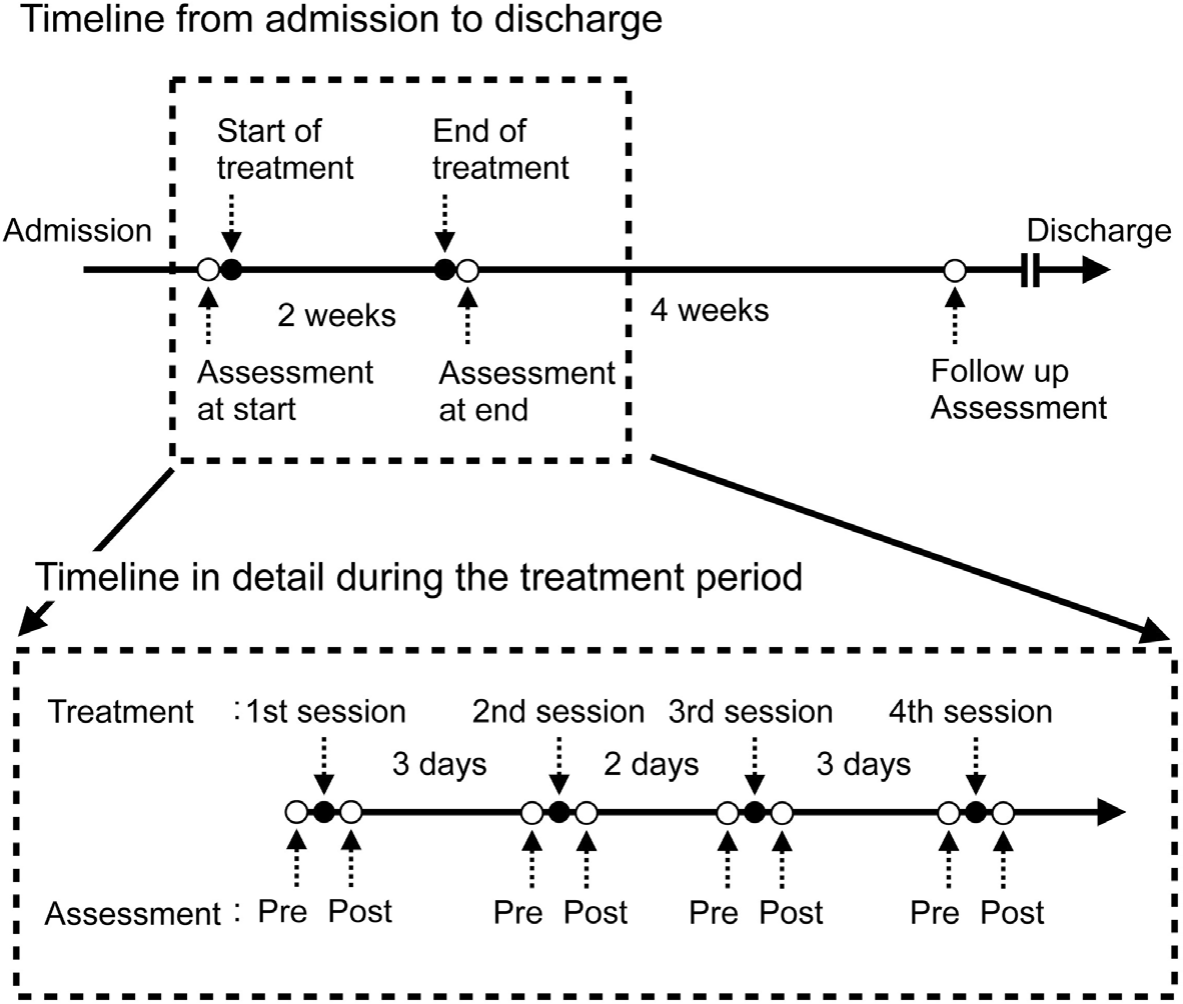
Timeline of the case. Timeline from admission to discharge and treatment details. Black circles indicate treatments; white circles indicate assessments.

**FIGURE 3:**
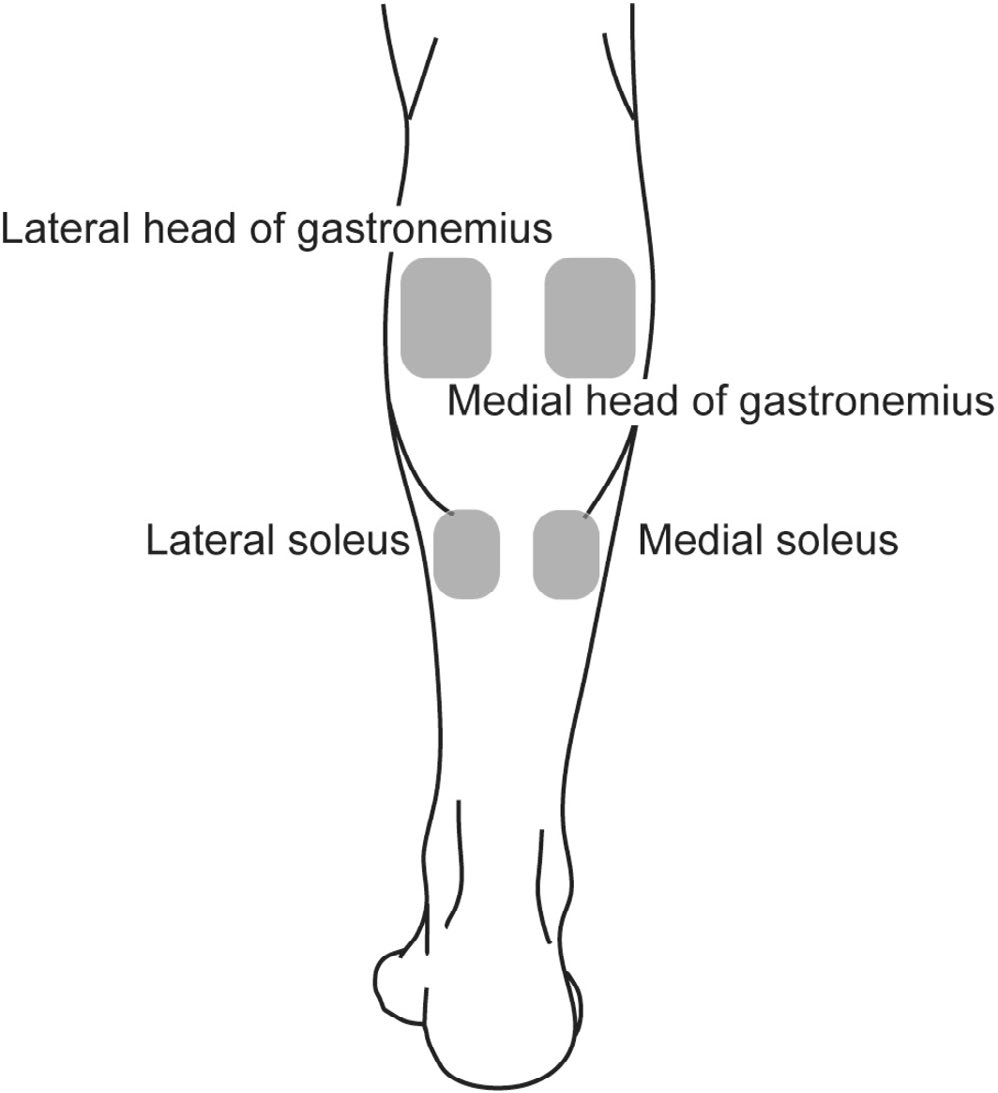
Locations of radial extracorporeal shock wave therapy administration on the triceps surae muscle.

The individual received 3 h of daily rehabilitation therapy, including physical, occupational, and speech and language therapies, in the rehabilitation ward. The primary goal of physical therapy was to achieve gait independence, with a focus on standing and walking training. After admission, spasticity gradually increased, leading to the initiation of antispastic medication (Lioresal 5 mg, three tablets per day) on day 11. However, as no significant improvement in spasticity was observed with the medication, spasticity treatment using an rESWT device (BTL‐6000 TopLine, BTL‐Japan) was initiated on day 18. rESWT was administered to four locations on the triceps surae muscle (medial and lateral heads of the gastrocnemius muscle and medial and lateral soleus muscles; Figure 2), with a total of 4000 pulses (1000 pulses per location). For the gastrocnemius muscle, the shockwaves were applied around the point of maximal muscle belly prominence and at the level of the musculotendinous junction between the triceps surae and the Achilles tendon for the soleus muscle. The stimulation intensity was set to 3.0 bar at a frequency of 10 Hz. The treatments followed a protocol previously shown to be effective in an individual with chronic stroke and were provided during physical therapy sessions twice weekly for 2 weeks, totalling four sessions (Figure 3 and Table 2) [15, 16, 19, 20, 21]. During the treatment period, physical therapy other than rESWT consisted of ROM exercises, standing exercises, and gait training.

**TABLE 2:** Protocols of radial extracorporeal shock wave therapy for lower‐limb spasticity in individuals with chronic stroke.

Study	Period of treatment		Parameters of intervention			Assessment time points	Effect duration
	Number of sessions	Frequency	Intensity (bar)	Frequency (Hz)	Number of shots		
Yoldaş [16]	4	Twice/week	2.0	10	1500	Immediately after final session; 4 weeks later	Immediate effect only; not sustained at 4 weeks
Wu [15]	3	Once/week	2.0	5	3000	1, 4, and 8 weeks after final session	Sustained at 1, 4, and 8 weeks
Radinmehr et al. [19]	1	—	1.0	5	2000	Immediately after session; 1 h later	Immediate effect up to 1 h
Radinmehr et al. [20]	1	—	1.0	5	2000	Immediately after session; 1 h later	Immediate effect up to 1 h
Nada et al. [21]	4	Once/week	2.5	4	1500	4 and 8 weeks after final session	Sustained at 4 and 8 weeks

## 4 Outcome and Follow‐Up

Assessments were performed before and after each of the four treatments and, again, 1 month after completion of the fourth treatment. The assessment indices included the MAS score of the triceps surae muscle, ROM of ankle dorsiflexion, Achilles tendon reflexes, and the clonus score. The Achilles tendon reflex is a reliable neurological assessment measure, with the following scale: 0 = reflex absent; 1+ = reflex small, less than normal, including a trace response or a response elicited only with reinforcement; 2+ = brisk, within the median normal range; 3+ = reflex enhanced, high normal or hyperreflexia; 4+ = reflex enhanced, more than normal, including intermittent clonus; and 5+ = sustained clonus [22]. The Achilles tendon reflex was assessed with the knee joint in slight flexion. The clonus score is a five‐point scale used to assess clonus caused by upper motor neuron dysfunction, based on duration: 0 = no clonus; 1 = 1–4 s; 2 = 5–9 s; 3 = 9–15 s; and 4 = more than 15 s [23]. The clonus score was assessed with the knee joint in mild flexion. The individual was seated in a wheelchair in a resting position during the assessments, which were conducted by the physical therapist.

**FIGURE 4:**
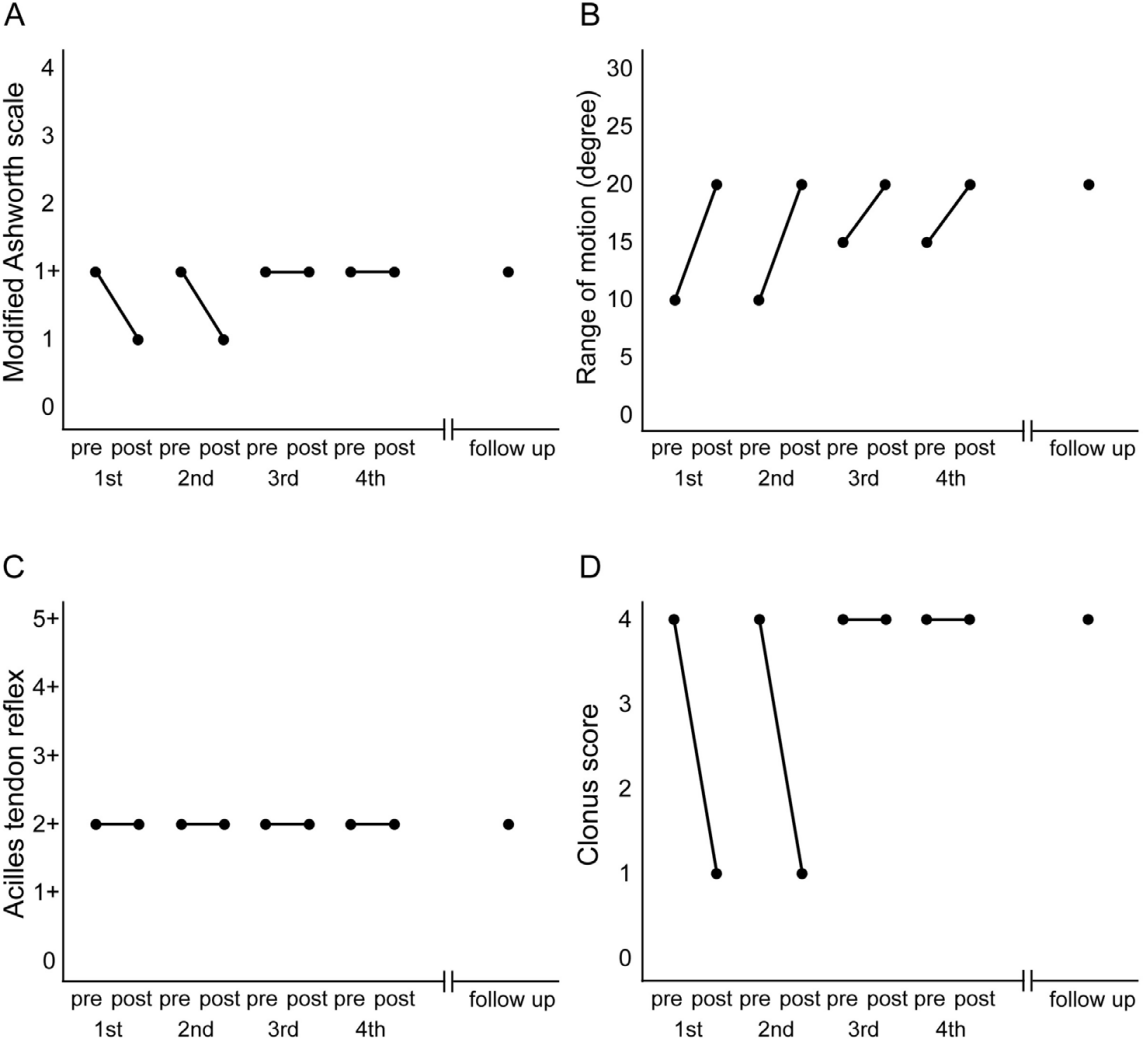
Change in indices before and after treatment from the first to fourth session. (A) Modified Ashworth Scale score. (B) Range of motion. (C) Achilles tendon reflex. (D) Clonus score.

Time‐course changes in the MAS scores, ROM, Achilles tendon reflexes, and clonus scores are shown in Figure 4. The MAS and clonus scores showed immediate improvement following the first and second treatments compared with pre‐treatment levels. However, no carryover effect was observed, as the individual reverted to the pre‐treatment state by the time of the next session. Follow‐up assessments conducted at the end of the fourth treatment and 1 month after completion showed no change from the pre‐treatment state. In contrast, the ROM showed immediate improvement after all treatments. Although no carryover effect was observed between the first and second treatments, it was observed between the second and third treatments. By the end of the fourth treatment and 1 month after its completion, the follow‐up assessment indicated a marked improvement compared with the pre‐treatment state. The individual successfully completed all scheduled rESWT sessions. Treatment adherence was confirmed by the attending therapist. No treatment‐related adverse events were observed.

The individual was discharged 112 days after symptom onset. At discharge, gait required minimal assistance with the use of an ankle–foot orthosis and a four‐point cane. Activities of daily living had significantly improved, with a total FIM score of 88 points—58 points for motor items and 30 points for cognitive items. The individual was discharged with preserved muscle tone and ankle dorsiflexion ROM following rESWT (Table 1).

## 5 Discussion

Four sessions of rESWT were administered to an individual with hemiparesis following a subacute stroke, and changes in the spasticity of the triceps surae muscle, along with other outcomes, were observed before and after treatment. In recent years, the importance of managing spasticity during the subacute phase of stroke has been increasingly emphasized [24, 25]. Common interventions during this period include stretching, appropriate positioning, oral antispastic medications, physical modalities such as electrical stimulation, and, in selected cases, botulinum toxin injections and intrathecal baclofen therapy [24, 25]. Several studies have reported that early and proactive management of spasticity during the subacute phase may help prevent its progression and the development of contractures [26, 27, 28]. However, the effects and potential cumulative benefits of novel interventions such as rESWT during this critical period remain poorly understood. To the best of our knowledge, this is the first report detailing the treatment course of multiple sessions of rESWT in an individual with stroke during the subacute phase.

Although the exact mechanisms by which radial shock wave therapy alleviates spasticity remain unclear, several neurophysiological and tissue‐level pathways have been proposed [29]. Neurologically, inhibition of neuromuscular transmission and reduced excitability of the alpha motor neurons may contribute to transient modulation of reflex activity [21, 29, 30, 31]. However, non‐neurological mechanisms have also been reported [29]. For example, increased production of nitric oxide may lead to improved local blood flow, thereby contributing to the reduction of muscle stiffness [21]. Additionally, the mechanical stimulation provided may promote structural reorganization of muscle fibers and connective tissues, resulting in improved viscoelastic properties [32]. These non‐neurological factors may play key roles in promoting long‐term therapeutic effects, such as sustained improvements in joint ROM and the prevention of contracture [32]. Although some studies have reported inconsistent or minimal effects of rESWT on reflexive components, growing evidence supports a more consistent and longer‐lasting impact on nonreflexive elements such as muscle stiffness and tissue elasticity [33, 34].

Although immediate improvements were observed in the MAS score, which assesses spasticity, and in the clonus score, which evaluates one of its reflex components, there was no sustained improvement in the assessments conducted after completion of all treatments and during follow‐up compared with the pre‐treatment state. Previous studies in individuals with chronic spasticity have reported that improvement in spasticity lasted up to 8 weeks [15] or did not persist beyond 4 weeks [16]. The duration of the effect varied across studies, leaving the exact duration of spasticity improvement unclear. However, in the present case, the duration of the spasticity‐reducing effect was only a few days, which is notably shorter than those reported in previous studies [15, 16]. This difference in outcomes may be attributable to the fact that the individuals in the aforementioned studies were in the chronic phase, with an average post‐onset duration of 56 and 35 months, respectively, whereas the present case involved an individual in the subacute phase, approximately 3 weeks post‐onset. Spasticity generally increases over time after stroke onset, with the most significant increase occurring between 1 and 3 months after onset, and, in some reports, peaking during the first month, suggesting that it increases during the subacute phase [35, 36]. In this case, the treatment period was approximately 1 month after onset, precisely when spasticity tends to increase. Therefore, it is likely that it was more difficult to sustain the improvements observed in the MAS than in the chronic phase. Furthermore, the relative contributions of reflexive and nonreflexive components of spasticity are considered to change over time following stroke [37, 38, 39]. The reflexive component, specifically the excitability of the stretch reflex, tends to peak during the subacute phase (1–3 months post‐onset) [38], whereas the nonreflexive component, derived from muscle and connective tissue alterations, becomes more prominent during the chronic phase [39]. As previously noted, rESWT is assumed to exert more sustained effects on the nonreflexive component [32]. Therefore, in the present case—occurring during the subacute phase, when the nonreflexive component may be less pronounced than in the chronic phase—the observed effects might have been less durable.

In contrast, the nonreflexive component of spasticity, as reflected in ROM, demonstrated a carryover and cumulative effect with repeated treatments, resulting in sustained improvement observed at the end of all treatments and during follow‐up assessments compared with the pre‐treatment state. The nonreflexive component—such as fibrosis of muscle fibers and connective tissue—tends to become more fixed and less reversible during the chronic phase [40]. In contrast, during the subacute phase, these changes are still considered reversible and may respond more favorably to treatment [28]. In the present case, repeated application of rESWT targeting the nonreflexive component during the subacute phase may have contributed to the observed sustained improvements. This outcome supports the concept of early intervention in spasticity management and aligns with previous findings emphasizing the greater efficacy of rESWT on nonreflexive mechanisms [24, 25, 33, 34].

The results of this study provide new insights into this topic. The duration of the effect on spasticity may be shorter in individuals with subacute stroke than in those with chronic stroke. In this case, the effect diminished within 2–3 days between treatments and returned to baseline. Repeated botulinum toxin therapy enhances cumulative effects and therapeutic efficacy [41]. Although botulinum toxin and shock wave therapies differ in the duration of their effects per session, repeated shock wave therapy may similarly increase therapeutic benefits. In this study, the treatment frequency of twice a week did not produce a cumulative effect in the subacute phase. It is necessary to examine whether a cumulative effect can be achieved by further increasing the treatment frequency, as in the case of chronic‐phase individuals. Regarding ROM, carryover and cumulative effects were observed with repeated treatments, complementing previous studies showing that multiple sessions are more effective than a single treatment.

This study has some limitations. First, it remains unclear whether the rESWT treatment protocol used is optimal. rESWT involves various parameters, such as stimulus intensity, number of shots, stimulation site, and frequency of stimulation. Higher stimulation intensities are reported to be more effective [42]. In this study, a stimulation intensity of 3.0 bar was used. RCTs examining the effectiveness of rESWT on the triceps surae muscle have applied stimulation intensities of 1.0–2.5 bar [15, 16, 19, 20, 21], and the stimulation intensity in this study was higher than those used in previous studies. Therefore, it is unlikely that insufficient stimulus intensity affected the results, although it remains unclear whether the intensity used in the present study was optimal. Regarding the stimulation site, stimulation of the muscle belly and the myotendinous junction has been attempted and reported to be equally effective [43]. In the present study, only the gastrocnemius and soleus muscle bellies were stimulated. All RCTs examining the effectiveness of rESWT in the triceps surae muscle have confirmed the effectiveness of stimulating only the muscle belly [15, 16, 19, 20]; thus, it was considered appropriate to stimulate only the muscle belly in this case. Second, there were no restrictions on other treatments for spasticity. The effectiveness of treatments such as botulinum toxin therapy, oral antispastic drugs, stretching, and electrophysical therapies (e.g., electrical and vibration stimulation) has been reported [44, 45]. However, the individual was taking antispastic drugs and performing stretching exercises during the rESWT treatment period; therefore, it was difficult to isolate the effects of rESWT from these other interventions. Third, this study used the MAS to evaluate spasticity. Although it is the most commonly used clinical index for evaluating spasticity, it has limitations in distinguishing between spasticity and contracture compared with the Modified Tardieu Scale (MTS); some authors suggest that it may overestimate the extent of spasticity [46, 47, 48]. In future studies, the MTS should be used alongside the MAS for evaluation. Fourth, assessments beyond spasticity‐related outcomes were limited in the present study. Reports suggest that rESWT may contribute to improvements in balance function and a reduction in fall risk through mechanisms involving proprioceptive modulation [49, 50, 51], and if rESWT does enhance balance or gait performance, these effects may have indirectly influenced follow‐up spasticity‐related measures. Future studies should therefore consider incorporating broader assessments, including proprioception, balance, and gait‐related outcomes.

## 6 Conclusion

Triceps surae muscle spasticity and other outcomes were evaluated before and after rESWT in an individual with hemiparesis following a subacute stroke, and improvements in spasticity and ROM were observed over time. The results indicated that the nonreflexive component, represented by ROM, showed long‐term improvement, whereas the reflexive component, as measured by the MAS and clonus scores, demonstrated only an immediate effect. It is necessary to examine whether a cumulative effect can be achieved by further increasing the treatment frequency. In addition, parameters other than treatment frequency may also need to be considered. Further clinical trials are required to determine the optimal treatment frequency.

## Author Contributions

**Daisuke Kato:** conceptualization, data curation, formal analysis, writing – original draft, writing – review and editing. **Satoshi Hirano:** conceptualization, project administration, supervision, writing – original draft, writing – review and editing. **Naoki Mori:** conceptualization, supervision, writing – original draft, writing – review and editing. **Shota Itoh:** data curation, writing – review and editing. **Toshiki Ito:** data curation, project administration, writing – review and editing. **Taiki Yokote:** supervision, writing – review and editing. **Hirofumi Maeda:** project administration, writing – review and editing. **Yohei Otaka:** conceptualization, project administration, supervision, writing – original draft, writing – review and editing.

## Funding

The authors have nothing to report.

## Ethics Statement

As this case report is based on clinical observations and does not involve experimental intervention or data collection beyond routine care, ethical approval was not required in accordance with national guidelines.

## Consent

Written informed consent was obtained from the individual for publication of the case details and any accompanying images.

## Conflicts of Interest

The authors declare no conflicts of interest.

## Supporting information


**Data S1:** Dataset generated and/or analyzed in this case report.

## Data Availability

All data generated and/or analyzed during this study are included in this article and its online Supporting Information (Supporting Information S1). Further enquiries can be directed to the corresponding author.
